# Comparative study of environmental pollutants bisphenol A and bisphenol S on sexual differentiation of anteroventral periventricular nucleus and spermatogenesis

**DOI:** 10.1186/s12958-019-0491-x

**Published:** 2019-07-10

**Authors:** Naham John, Humaira Rehman, Suhail Razak, Mehwish David, Waheed Ullah, Tayyaba Afsar, Ali Almajwal, Iftikhar Alam, Sarwat Jahan

**Affiliations:** 1 Reproductive Physiology Lab, Department of Animal Sciences, Quaid- i- Azam University Islamabad, Islamabad, 45320 Pakistan; 2 Department of Community Health Sciences, College of Applied Medical Sciences, King Saud University, Riyadh, Kingdom of Saudi Arabia

**Keywords:** Endocrine disruptor, Bisphenol A, Bisphenol S, Immunohistochemistry, AVPV

## Abstract

**Background:**

Bisphenol A is well known endocrine-disrupting chemical while Bisphenol S was considered a safe alternative. The present study aims to examine the comparative effects of xenobiotic bisphenol-A (BPA) and its substitute bisphenol-S (BPS) on spermatogenesis and development of sexually dimorphic nucleus population of dopaminergic neurons in the anteroventral periventricular nucleus (AVPV) of the hypothalamus in male pups.

**Methods:**

Sprague Dawley rat’s pups were administered subcutaneously at the neonatal stage from postnatal day PND1 to PND 27. Thirty animals were divided into six experimental groups (6 animals/group). The first group served as control and was provided with normal olive oil. The four groups were treated with 2 μg/kg and 200 μg/kg of BPA and BPS, respectively. The sixth group was given with 50 μg/kg of estradiol dissolved in olive oil as a standard to find the development of dopaminergic tyrosine hydroxylase neurons in AVPV regions. Histological analysis for testicular tissues and immunohistochemistry for brain tissues was performed.

**Results:**

The results revealed adverse histopathological changes in testis after administration of different doses of BPA and BPS. These degenerative changes were marked by highly significant (*p* < 0.001) decrease in tubular and luminal diameters of seminiferous tubule and epithelial height among bisphenols treated groups as compared to control. Furthermore, significantly increased (p < 0.001) TH-ir cell bodies in the AVPV region of the brain with 200 μg/kg dose of BPA and BPS was evident.

**Conclusion:**

It is concluded that exposure of BPA and BPS during a critical developmental period can structural impairments in testes and affects sexual differentiation of a dimorphic dopaminergic population of AVPV region of hypothalamus in the male brain.

## Background

Bisphenol A (BPA) is employed in industry, particularly in polycarbonate plastics industrial processes and foodstuff containers. The resiliency of BPA plastics has led to their use in medical equipment’s such as heart-lung machines, incubators, hemodialyzers, and dental sealants and fillers; also, their light weight and optical clarity have made them especially useful for eyeglasses. Phthalates help make plastic, like pacifiers, flexible. Due to his widespread applications, the use of BPA has gathered cumulative consideration over the last decade, particularly in terms of human safety. It is believed that both BPA and phthalates can leach from the plastic into food, liquid, and directly into the mouths of children while sucking on pacifiers or teethers. It has been estimated that levels of conjugates of BPA in urine are above safety thresholds in 90% of individuals tested in several population studies [1]. Fetal exposure to high doses of dibutyl phthalate was shown to cause a testicular dysgenesis syndrome (TDS)-like phenotype in the rats [2]. TDS is a male reproduction-related condition characterized by the presence of symptoms and disorders such as hypospadias, cryptorchidism, poor semen quality, and testicular cancer. TDS is a result of disruption of embryonal programming and gonadal development during fetal life [2] Various reports established that BPA acts as an endocrine disrupting chemical and its exposure can affect the reproductive system of a male by disturbing spermatogenesis and fertility, furthermore, its exposure during development causes organizational effects on brain [3–10]. BPA has increased excess to estrogen sensitive tissues in brain [16]. At the hypothalamic or pituitary level, BPA may inhibit the estrogen binding to its receptors. Thus, circulating estrogen reduces its negative feedback actions on luteinizing hormone (LH) and follicle stimulating hormone (FSH) release, resulting in high levels of circulating LH and FSH. Thus, it is suggested that BPA’s actions are greater during development[13–15].

Bisphenol S (4, 4′–dihydroxydiphenyl sulphone) abbreviated as BPS, is a man made, industrial chemical and another member of bisphenol family. BPS has increased stability and resistance against sunlight and high temperatures. Like BPA, BPS is an endocrine disrupting molecule and its increasing use is alarming for human health [11]. In zebrafish, BPS exposure has shown to induce a reduction in gonadal weight, alteration in hormonal and disruption in the normal process of reproduction (i.e. decreases egg production and hatchability, increases embryo malformations, increase in time to hatch) [17]. BPS exposure also decreases body length, increases male and female sex ratio and causes reproductive disruption, disturbs the balance of sex steroid hormones in adult zebrafish [18]. Several studies show androgenic and anti-androgenic activities of BPS [19]. Similar to BPA, BPS can induce alterations in an embryonic, nervous and endocrine system [17, 20, 21]. Exposure of BPA and BPS to zebrafish embryo causes 180 and 240% increase in hypothalamic neurogenesis [21]. During early developmental stages, BPA exposure has been suspected to affect testicular development and spermatogenesis [22, 23].

In mammals, ovaries are generally quiescent in developing females, so reproductive tract and brain development occurs in the absence of estrogen. Whereas in developing male, elevated estrogen levels locally synthesized by aromatization of testosterone (testicular) are present. The sexual differentiation of estrogen exposure and hormonal synthesis results into distinct development of neuroanatomical circuits, neuroendocrine functions, and reproductive behaviors in both male and females [24]. The anteroventral periventricular nucleus (AVPV) is a small cluster of neurons along the wall of the third ventricle just caudal to the vascular organ of lamina terminalis (OVLT). AVPV receives sexually dimorphic innervation by dopaminergic afferents that regulate gonatropin-releasing hormone and sexual reproduction [25, 26]. AVPV is three times more sexually dimorphic in females, being larger in volume and containing more cells in females than male. This suggests that this brain region is very important in controlling the estrous cyclicity in females. In female AVPV, for dopamine synthesis, the rate limiting enzyme tyrosine hydroxylase (TH) expressing neurons are more abundantly and topographically distributed and are distinct from males [26, 27]. The perinatal administration of testosterone or estrogen can defeminize the neuron count and distribution in female AVPV [28]. One of the function of TH cells of AVPV is to regulate the secretion of gonadotropin releasing hormone (GnRH) neurons in the medial preoptic area (POA) [29]. Total numbers of TH-cells are more in female’s AVPV than males, hence sexually dimorphic GnRH secretions. Estradiol administration have no effect on TH cell numbers in males and it is suggested that BPA have antiestrogen action on this neural cell population [24].

If it is speculated that BPA acts as estrogen or an anti-estrogen in the AVPV region, then it can be hypothesized that more or less sexually dimorphic areas could be affected by exposure to BPA or BPS. Considering our chosen endocrine active compounds to be estrogenic, masculinization of female AVPV should be evident via a reduction in TH-expressing neurons number. In contrast, anti-estrogenic bisphenols through increment in TH-expressing neurons count should de-masculinized the male AVPV.

This study was designed to determine the low dose effect of BPA and BPS on sexual differentiation of the AVPV region of the male brain at the neonatal stage and to find out the possible role of endocrine disrupting chemicals on the reproductive system of male rats at initial stages of life. These finding will further provide the evidence of negative effects of BPA and its analogue BPS on the reproductive system of male rats.

## Methods

### Animals

At the start, five wooden breeding cages were separated and five adult female Sprague Dawley rats were kept in each breeding cage with two adult male Sprague Dawley rats. Ten days later, adult male rats were separated from females. Laboratory pelleted food and water was available to animals ad libitum. Pregnant females were reared singly till the birth of pups on day 22 (gestational day-GD-22). The day of birth of the pups was called as postnatal day 1 (PND1. Total numbers of pups were counted and separated from female pups by measuring the anogenital distance (AGD) under a stereomicroscope. For each experimental group, six male pups were separated from each litter. Animals were handled and sacrificed according to the guidelines provided by the Ethical Committee of Animal Sciences department, Faculty of Biological Sciences, Quaid-i-Azam university, Islamabad.

### Experimental plan

Newborn pups (PND1) animals were distributed into six groups (*n* = 6/group). The first group served as control and received subcutaneous injection of olive oil (50 μl). The second and third group rats were treated with BPA (2 μg/kg and 200 μg/kg) respectively. Fourth and fifth group male pups were injected with BPS (2 μg/kg and 200 μg/kg) respectively. The sixth group was given with 50 μg/kg of estradiol dissolve in olive oil as a standard to a find development of dopaminergic tyrosine hydroxylase neurons in AVPV regions. All treatments were dissolved in olive oil and were given from PND 1 to PND 27 with reference to prior studies [30–32]. Estradiol treated group was only used to serve as a standard to find a development of dopaminergic tyrosine hydroxylase neurons in AVPV of rodents’ hypothalamus. All dosages were adjusted daily according to body weight. We selected a higher dose of 200 μg/kg because it is higher suspected human exposure dose and a low dose of 2 μg/kg approaches to human exposure level [24, 33].

On PND 27, animals were weighed (using Sarotoreious Digital Balance) and sacrificed by decapitation. The brain tissues were used only for immunohistochemistry. Testicular tissues were dissected out for tissue histology and were washed in saline and weighed. Other tissues (brain, kidney, liver, and intestine) were also dissected out and weighed also.

### Histological analysis

Histological analysis was done exactly by using a method given by Ullah et al. [34]. Firstly, testis was kept in sera (composed of ethyl alcohol, formaldehyde, and glacial acetic acid in a ratio of 6:3:1), and then placed in 10% formalin for 24 to 48 h. Following fixation, tissues were dehydrated with ascending grades of ethanol, cleared with xylene and embedded in paraffin wax. Microtomy was then carried out and seven μm thick sections of testis were cut (Thermo, Shandon finesse 325, UK). Testis sections were then fixed on albumenized glass slides, placed in an incubator overnight for completion of deparaffinization and on next day, were stained with hematoxylin and eosin and then, observed under the light microscope (Nikon, 187,842, Japan). Leica LB microscope (Germany) equipped with canon digital camera was used for microphotography. For histolomorphometric studies, Image J software was used for the measurement of testis parameters (National Institutes of Health, Bethesda, MD, USA).

### Brain tissue fixation and processing

The entire hypothalamic blocks of the brain were placed in 4% paraformaldehyde overnight. Next day, these samples were dehydrated and cleared through different grades of ethanol and xylene follow embedding in paraffin wax. The tissue was then deparaffinized with xylene and rehydrated in graded ethanol before being washed with twice-distilled water. Later, hypothalamic blocks were cut into consecutive sections of 7–10 μm thickness on a cryostat (Bright OTF 5000, A-M Systems, Sequim, Washington, USA; temperature − 25 °C) and preserved in an antifreeze cryoprotectant solution (1% polyvinylepyrrolidone, 30% ethylene glycol and 30% sucrose in PBS) at − 20 °C until used for immunocytochemistry.

### Immunocytochemistry

Standard double immunocytochemistry protocol was followed for the processing of hypothalamic sections. Total numbers of TH cell bodies were immunolocalized in the mediobasal hypothalamic region using a cocktail of primary antibodies directed against TH. For TH expression monoclonal antibody TOH A1.1 raised in mouse against human TH (Catalogue no. ab-150,659; Abcam Biotechnology, Inc., Cambridge, United Kingdom) was used and Alexa Flour 488 labeled goat anti-mouse IgG (Catalogue no. ab150117; Abcam, Cambridge, UK) was used as a secondary antibody. From each animal, three slides of hypothalamus were obtained for TH labeled immunostaining. While, one section from each group was used as primary antibody omitted control. Hypothalamic sections were washed with phosphate buffered saline (PBS; PH7.3) (Omnipur PBS tablets, Calbiochem, EDM chemicals Inc., Gibbstown, New Jersey, USA) for 8 × 15 minutes at room temperature (25 °C), to remove the cryoprotectant afterwards sections were incubated in incubation solution containing 10% normal goat serum, 0.05% Triton-X 100 and 0.1% bovine serum albumin (BSA) in PBS for two hours on shaker at room temperature to block the non-specific binding of the antibodies. Sections were then washed with PBS for 3 × 15 minutes. Then sections were incubated at 4 °C for 48 h on a shaker in a cocktail of primary antibodies (anti-TH antibody at 1:20) diluted at PBS containing 10% normal goat serum, 0.03% Triton X- 100 and 0.5 BSA. Control sections were incubated in PBS with 10% normal goat serum in PBS containing 0.05% TritonX-100 and 0.1% BSA. After incubation of 48 h, sections were washed in PBS for 3 × 15 min at room temperature. After washing, the sections were incubated in the cocktail of secondary antibodies Alexa Flour 488 labeled goat anti-mouse at 1:1000 diluted in PBS containing 0.05% Triton X- 100 and 0.1 BSA 10% normal goat serum for 2 h in dark, at room temperature on a shaker. Control sections were also incubated with secondary antibodies at this stage. After incubations, sections were again washed with PBS 3 × 15 min. Later, all sections were mounted on super frosted glass slides (Micro slides, Santa Cruz Biotechnologies, Dallas, Texas USA) and left overnight for drying at 4 °C in dark. Then coverslip was placed on slides using laboratory-prepared gelvatol as a mounting medium. Gelvatol was prepared by adding 10.5 g polyvinyl alcohol and few crystals of sodium azide in 12 ml glycerol. Then 21 ml distilled water and 53 ml Tris (Ph 8.5) were added. The mixture was stirred on low heat for six hours until reagents were properly dissolved. The mixture was placed overnight in a refrigerator and centrifuged at 5000 g for 15 minutes. Slides were stored at 4 °C after drying until further analysis. Slides were viewed using fluorescent microscope (Bx51, Olympus, Tokyo, Japan) to localize, Tyrosine hydroxylase immuno-reactive neurons in the AVPV region of the brain. Sections were examined at 10X, 20X and 40X magnifications.

### Statistical analysis

For histological data analysis of testes and the number of single TH-immunoreactivity cells, GraphPad prism 5 software (GraphPad Software, Inc., San Diego, CA, USA) was used. One way analysis of variance (ANOVA) was used for statistical analysis of studied parameters. Later, Dunnet’s multiple comparison tests were practiced to relate the controls results with treated ones. All the data is shown as mean ± SEM. Significance value was set at *p* < 0.05.

## Results

### Effect of subcutaneous exposure of bisphenol A bisphenol S on body weight (g) in male rats, during the neonatal period

Mean ± SEM body weight of all experimental groups in male rats are shown in Table 1. As compared to control group, significant (p < 0.05) change was detected in body weight recorded on PND 8, among low and high concentrations of BPA groups. On PND 16, very remarkable increment (*p* < 0.001) was noticed in body weight among BPA 2 μg/kg and BPS 2 μg/kg treated groups comparison with control. Animals treated with BPS 200 μg/kg showed significance change (*p* < 0.05) than control group. BPA (2 μg/kg dose) induced significant (*p* < 0.01) increase in body weight on PND 24 compared to the control group.

**Table 1:** Comparative effects of different concentrations (2 μg/kg, 200 μg/kg) of Bisphenol A and Bisphenol S on body weight (g) recorded on 8th, 16th, 24th and 28th day of development

Groups	Day 8	Day 16	Day 24	Day 28
Control	9.71 ± 0.52	12.4 ± 0.87	20.5 ± 0.92	43.7 ± 6.54
BPA (2 μg/kg)	13.6 ± 0.65	21.9 ± 0.32 a**	35.3 ± 0.60 a**	40.1 ± 1.66
BPA (200 μg/kg)	13.0 ± 0.30	12.8 ± 0.87	19.4 ± 1.88	37.6 ± 3.38
BPS (2 μg/kg)	11.5 ± 0.28	19.9 ± 1.50 a**c***	28.9 ± 2.79	40.4 ± 0.32
BPS (200 μg/kg)	13.9 ± 0.38	17.2 ± 0.41 a*	31.6 ± 2.12 a*c***	38.5 ± 2.05

Values are expressed as mean SEM *p < 0.05, **p < 0.01, p*** < 0.001

a = Values vs control, b = Values vs Bisphenol A 2 μg/kg, c = Values vs Bisphenol A 200 μg/kg, d = Values vs Bisphenol S 2 μg/kg

### Effect of subcutaneous exposure of BPA and BPS on weight of testis (g), prostate (g), seminal vesicle (g), liver (g), heart (g) and kidney (g)

Table 2 indicated the effect of various treatments of BPA and BPS on organ weights of rats.

**Table 2:** Comparative effects of different concentrations (2 μg/kg,200 μg/kg) of Bisphenol A and Bisphenol S on body organs weight

Groups	Testes paired weight(g)	Prostate (g)	Seminal Vesicle (g)	Liver (g)	Heart (g)	Kidney (g)
Control	0.74 ± 0.19	0.04 ± 0.01	0.34 ± 0.19	1.90 ± 0.20	0.58 ± 0.22	0.59 ± 0.21
BPA 2 μg/kg	0.24 ± 0.01a*	0.06 ± 0.01	0.03 ± 0.01	1.90 ± 0.17	0.20 ± 0.01**	0.45 ± 0.01
BPA 200 μg/kg	0.39 ± 0.17	0.05 ± 0.01	0.01 ± 0.0	1.92 ± 0.08	0.23 ± 0.02**	0.49 ± 0.02
BPS 2 μg/kg	0.25 ± 0.01	0.02 ± 0.01b**	0.09 ± 0.07	1.63 ± 0.13	0.23 ± 0.01**	0.55 ± 0.00
BPS 200 μg/kg	0.24 ± 0.03	0.02 ± 0.01b**	0.02 ± 0.01	1.43 ± 0.15	0.23 ± 0.02**	0.47 ± 0.02

Values are expressed as mean SEM

*p < 0.05, **p < 0.01, p*** < 0.001

a = Values vs control, b = Values vs Bisphenol A 2 μg/kg, c = Values vs Bisphenol A 200 μg/kg, d = Values vs Bisphenol S 2 μg/kg

Testicular weight seen to be increased (p < 0.05) in BPA 2 μg/kg, BPS 2 μg/kg and BPS 200 μg/kg treated groups than the control. No significant differences were noticed in prostrate, seminal vesicle, liver and kidney weights when all experimental groups were compared to control. Heart weight of BPA and BPS 2 μg/kg and 2 μg/kg treated animals show significance change (p < 0.01) as compared to control.

### Tissues histology

The histological studies of the testis showed closely arranged seminiferous tubules and normal spermatogenesis in the control group. A photomicrograph of a section of the testis of 27 days old albino rat of the BPA 2 μg/kg group showing a prominent increase in the interstitial spaces between seminiferous tubules appeared irregular and smaller in size as compared to control group. A decrease was observed in the epithelial diameter so it is evident that there will be a reduction in the number of spermatogonia and primary spermatocytes near the lumen of the tubule. Some germ cells appeared shattered and separated with the appearance of empty spaces; some appeared desquamated toward the lumen with the disappearance of the early spermatids. Lumen diameter in BPA 2 μg/kg treated group was increased as compared to the control group. BPA 200 μg/kg treated rat testis showing a normal testis structure with a slight decrease in tubular diameter as compared to BPA 2 μg/kg. There was an increase in the interstitial spaces between the tubules with a slight appearance of destructed Leydig cells. BPA 200 μg/kg treated rats showed less loss of stratification and disorganization of the lining epithelium of the seminiferous tubules show more closely resembled to control group. Epithelial layer contained spermatogonia, primary spermatocytes, and early spermatids. Some interfollicular spaces of the tubules showed destructed interstitial tissue and most sections of testis treated with BPA 200 μg/kg shows a reduction in lumen diameter show slight variation as compare to control group. Histomorphology of the testicular section from BPS 2 μg/kg showed a major difference in the appearance of seminiferous tubules compared to control group. Lumen diameter is somehow appeared similar to the control group.

### Seminiferous tubule diameter

**Fig. 1:**
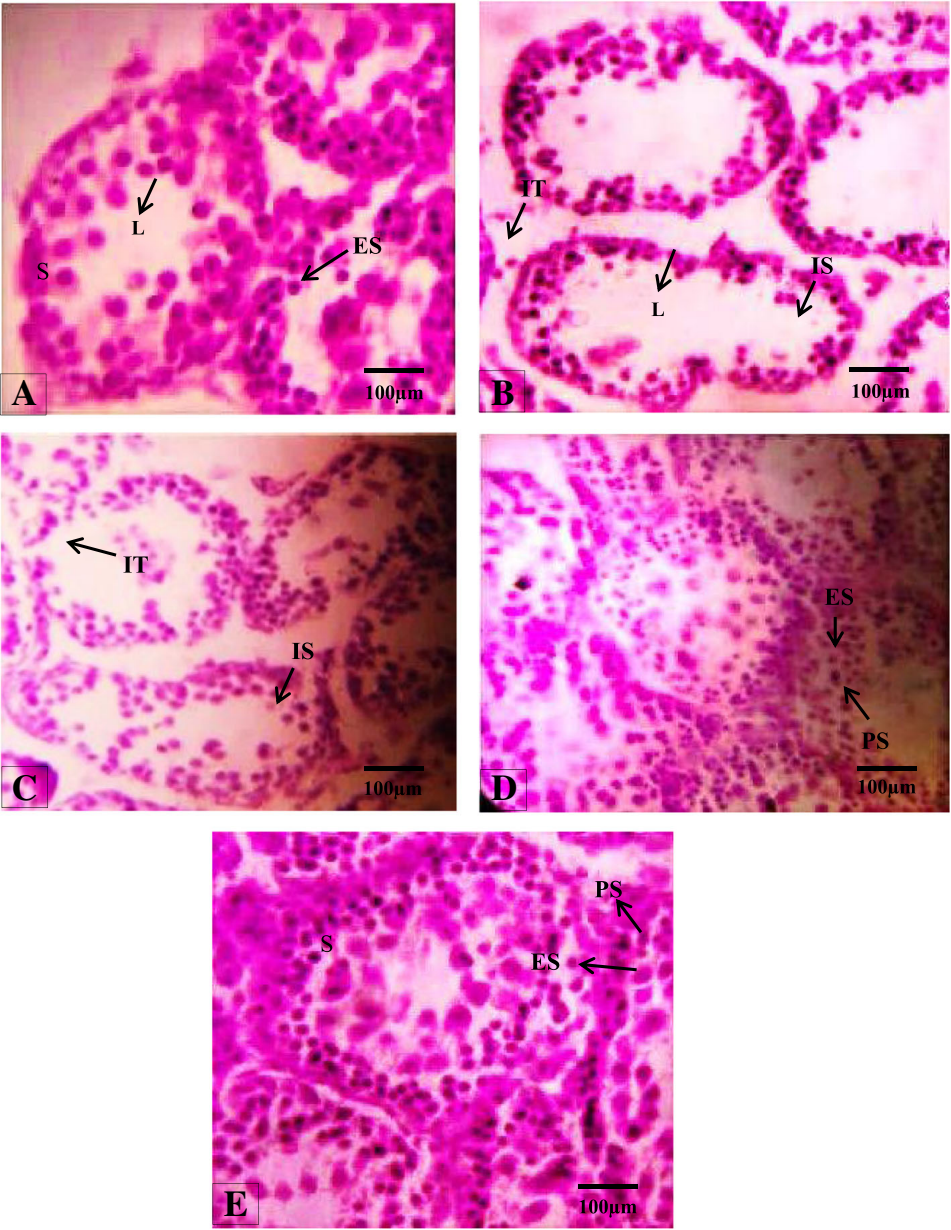
Photomicroghaph of 27 days old neonatal male rat seminiferous tubules(H&E, 40X) from: (**a**) Control; showing normal morphology of closely packed tubules with basal lamina, stratification and increased spermatogenic epithelium Spermatogonia, primary spermatocytes and early spermatids well developed lumen, (**b**) BPA group treated with 2 μg/kg of dose; showing noticeable increase in interstitial spaces between destructed tubules and basal lamina, decrease in epithelial height, only spermatogonia and large lumen, (**c**) BPA group treated with 200 μg/kg; showing decreased tubular diameter damaged interstitial tissues and increased interstitial spaces, narrow lumen, (**d**) BPS 2 μg/kg treated group showing semineferious tubules without interstitial space, minimal damage to epithelial, decrease in lumen diameter, (**e**) BPS 200 μg/kg; showing no interstitial space, normal spermatogenesis and basal lamina around tubules and very short lumen with early spermatids. Interstitial space (IS), Spermatogonia(S), Primary spermatocytes (PS), Early spermatids (ES), Epithelial Height (EH), Interstitial tissues (IT), Basal lamina (BL), Lumen (L)

Significantly increased (*p* < 0.001) diameter was observed when BPA 2 μg/kg and BPS 200 μg/kg treated groups compare with a control group. No significant difference was found when compared BPS 2 μg/kg treated group with BPA 200 μg/kg treated groups as compared to control. (Table 3, Fig. 1).

**Table 3:** Mean ± SEM of seminiferous tubule diameter, tubular lumen diameter, seminiferous tubule epithelial height, tunica albuginea height of testis in control and treated groups after 28 days of treatment

Groups		Seminiferous Tubule Diameter	Lumen Diameter	Epithelial Height	Tunica Albuginea Height
	Control	106.46 ± 2.94	84.19 ± 3.46	35.59 ± 0.63	19.79 ± 1.14
	BPA 2 μg/kg	126.03 ± 4.19^a^***	61.72 ± 3.09 ^a^***	23.00 ± 0.48 ^a^***	16.34 ± 0.54
	BPA 200 μg/kg	133.98 ± 3.23^a^***	71.22 ± 2.60 ^a^**	25.16 ± 0.48^ab^***	17.66 ± 0.90
	BPS 2 μg/kg	106.60 ± 2.46^bc^***	62.90 ± 1.77 ^a^***	32.39 ± 0.54^abc^***	18.61 ± 0.63
	BPS 200 μg/kg	114.29 ± 2.45^c^***	64.86 ± 2.20 ^a^***	26.55 ± 0.57^abd^***	18.19 ± 0.67

Values are expressed as mean SEM

*p < 0.05, **p < 0.01, p*** < 0.001

a = Values vs control, b = Values vs Bisphenol A 2 μg/kg, c = Values vs Bisphenol A 200 μg/kg, d = Values vs Bisphenol S 2 μg/kg

### Tubular lumen diameter

Mean tubular lumen diameter in BPA treated animals was decreased significantly (*p* < 0.001) as compare to control animals. Statistical difference in the mean of the lumen diameter was decreased significantly (*p* < 0.01) when compare BPA 200 μg/kg treated group with a control group. A highly significant decrease (*p* < 0.001) in tubular lumen diameter was observed in BPS 2 μg/kg and BPS 200 μg/kg treated groups respectively when compared to control group (Table 3, Fig. 1).

### Epithelial height

Mean epithelial thickness was decreased significantly (*p* < 0.001) in BPA 2 μg/kg treated group as compare to control group. Highly significant (*p* < 0.001) reduction was seen in BPA 200 μg/kg treated group as compare to control group. There was highly significant (*p* < 0.001) decrease in mean was observed between BPS 200 μg/kg treated group and BPS 2 μg/kg treated group as compare to control group (Table 3, Fig. 1).

### Tunica Albugenia height

There was no significant change was noticed in Tunica Albuginea height among all treated groups except BPA 2 μg/kg showed significant decrease (*p* < 0.005) with their comparison to that of the control group (Table 3, Fig. 1).

### TH immunoreactive cells

**Fig. 2:**
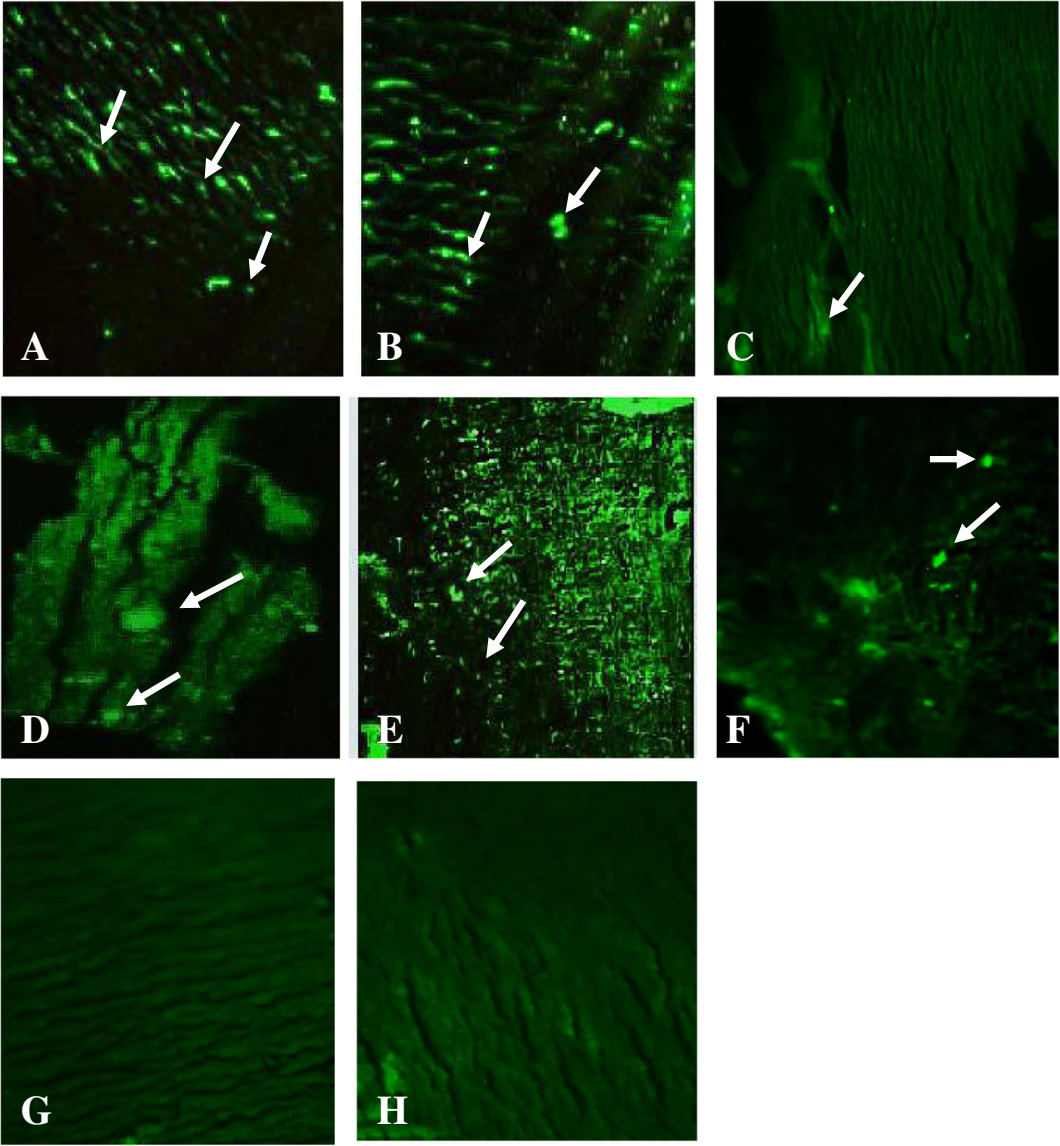
Photomicrograph (10X) of TH-immunoreactive cells in AVPV of representative Sprague Dawley male rat pups that were neonatal treated with BPA 2 μg/kg (**a**), BPA 200 μg/kg (**b**), BPS 2 μg/kg (**c**), BPS 200 μg/kg (**d**), Control (**e**), Estradiol (**f**). Omitted controls **g** and **h** show no significance results

TH expression is sexually dimorphic in the AVPV region of brain. No significant alterations were observed in TH-ir cell number between control, estradiol and a low dose of BPA 2 μg/kg treated groups. Extremely prominent (*p* < 0.001) rise was seen in the TH-ir cell bodies between BPA 200 μg/kg treated group and control group. Treatment with a low dose of BPS 200 μg/kg and a high dose of BPS 2 μg/kg were significantly (*p* < 0.001) different from control group (Table 4, Fig. 2).

**Table 4:** Comparison of Mean ± SEM TH-ir neuronal cells of control, estradiol, BPA 2 μg/kg, BPA 200 μg/kg, BPS 2 μg/kg, BPS 200 μg/kg treated groups, in 20 μm thick hypothalamic sections of 28 days old male rats

Animals	Control	Estradiol 50 μg/kg	BPA 2 μg/kg	BPA 200 μg/kg	BPS 2 μg/kg	BPS 200 μg/kg
1	74.5	60.75	75	163.75	183	148
2	46.25	56.25	101	178.25	100.75	119.5
3	65.25	56.5	94.75	56.5	64.5	87
TH-ir Cell Bodies	62 ± 4.47	57.83 ± 3.68	90.25 ± 7.41^b*^	175 ± 8.2^abc***^	80.08 ± 10.03^d***^	118.16 ± 10.23^abd***e*^

Values are expressed as mean SEM

*p < 0.05, **p < 0.01, p*** < 0.001

a = Values vs control, b = Values vs Bisphenol A 2 μg/kg, c = Values vs Bisphenol A 200 μg/kg, d = Values vs Bisphenol S 2 μg/kg

## Discussion

Over the last decade, it has become well known that BPA, a ubiquitous environmental endocrine disruptor administration causes reproductive toxicity and gonadal damage [35–39]. Similarly, BPS is another strong estrogenic and anti-androgenic compound, which has been banned now in different countries due to its toxic effects on the physiology of the reproductive system [19, 40, 41].

The present study shows irreversible organizational effects in testis of prepubertal male rats exposed to BPA and BPS different concentrations (2 μg/kg and 200 μg/kg) during the neonatal stage [42]. We observe no significant change in final body weight in all treatment groups. These results are similar to a study where animals were treated with 50 μl genistein and 5 μg/kg BPA (Gen/BPA 0.005) and no significant change in body weight were observed [43]. Alike results were experienced in postnatal male mice as compared to control mice when administered with BPA at early embryonic stage [36]. Ullah et al. (2016) also noticed no alteration in body weight of adult male rats after sub-chronic oral administration of BPS [34]. However, in the current study significant increase in body weights was recorded at PND 16 and PND 24 in BPA (2 μg/kg) and BPS (2 μg/kg and 200 μg/kg) treated groups. Our results are in accordance with the former study by Rubin et al. (2001) where female rat dams, when exposed to BPA from day 6 of pregnancy until lactation, resulted in increased body weight relative to control group in offspring [45]. Previously, Sakaue et al. reported that exposure of BPA (2 μg/kg to 200 μg/kg) to adult male rats resulted in decreased testicular weight and impaired spermatogenesis [44].

It is known that administration of exogenous estrogen results in reduced adipocyte number and it might contribute to reduction of body weight [46–50]. No significant change was seen for liver, kidney and heart weights when compared in experimental groups (Table 2). Some studies showed no significant change in organ weights by BPA administration [39].

In the present study, pubertal spermatogenesis for histomorphometric analysis was characterized by seminiferous tubule diameter, lumen diameter, epithelial and tunica albugenia height. A significant increase was witnessed in interstitial spaces and tubular lumen diameter with exposure to bisphenol analogues. Both BPA low and high dose exposure led to reduce seminiferous tubule diameter, epithelial and tunica albugenia height and germ cells. In our former study by Jahan et al., 2016, it has been reported that administration of BPA in adult rats induces similar changes with low efficiency of spermatogenesis. Studies have shown that not only tissue morphology is affected by BPA but also the number of mature spermatids becomes limited [34]. Similarly, low dose exposed BPS groups underwent a minimal level of damage to epithelial and reduction in lumen diameter, however, seminiferous tubule presented almost same pattern as control and as such no evident damage in high dose BPS exposed group.

In vertebrates and mammals, reproductive system and brain are physiologically and anatomically differentiated in both males and females, as a result of postnatal sex steroids during early development in the hypothalamic sexually differentiated anteroventral periventricular nucleus (AVPV). AVPV region of the brain has multiple cell types that are sexually dimorphic and tyrosine hydroxylase positive cells are one of them [47]. Aromatization of estrogen in the medial region of male brain results in expression of few Tyrosine hydroxylase cell. It is well documented that in rats and mice, neurons of TH + ve cells in the AVPV of brain act as a potent anatomical marker of brain sexual differentiation [48, 49]. TH neurons number in the AVPV region of brain is effectively reduced by administrating testosterone or estradiol at the perinatal and/or postnatal stage. No sex difference in TH number was seen in ERKO (lack ER) mice, however, mice lacking TH receptor for androgen maintain sexual dimorphism in TH number [49]. So through these studies, it might be proposed that ER is important for estrogen action in reducing TH neuron number in males as compared to females.

The postnatal exposure of bisphenol analogues has displayed no significant effect at low dose 2 μg/kg of BPA and BPS on the total amount of TH-ir neurons in males. Our present results are in line with findings of Patisaul et al. (2006) [24]. But the significant increase was observed in the number of TH-ir cell bodies with high doses of 200 μg/kg of BPA and BPS. These results indicate that BPA interferes with estrogen action at low dose [28] and acts as antiestrogenic at high doses resulting in demasculinization of positive cells that can lead to reproductive damage in adulthood. The data point that just like BPA, BPS effectively interferes with endogenous estrogen and demasculinizes expression of TH in AVPV region suggesting that BPA, as well as BPS, play an anti-estrogenic role in the developing brain of male rats. Various scientists also support this idea [24].

The underlying mechanisms behind estrogen actions on TH expression patterns in AVPV in the neonatal males are not well understood. But it appears to be acting through estrogen receptor signaling. According to Rubin et al. (2011), a study conducted on the pregnant mice delivering low levels of BPA to their offspring by subcutaneous implanted Alzet pumps resulted into decreased TH cell number in AVPV of females as compared to control male [12]. Simerly et al., (1997) reported that male mice in which TH-ir neurons lack ERa, are phenotypically similar to wild type female mice, suggesting that ERa is important for the normal masculinization of the male brain by estrogen [44–49].

It is also demonstrated that developing AVPV TH-ir cells are sensitive to EACs disruption, reliability of dopaminergic TH expression patterns make it more vulnerable to examine the sensitivity of EACs in the neonatal brain [50]. Previous epidemiological studies for early life exposure of BPA in young girls in humans associated with a high level of anxiety and hyperactivity suggest that early life BPA exposure has injurious, sex specific and neural effects in humans [51, 52, 56].

According to observations of the USA population from 2010 to 2014, a trend of using BPS as BPA substitute has led to increased use of this chemical in industrial products [53]. Sexual dimorphism in AVPV volume and TH neurons are appeared to be influenced by BPA exposure and there are different mechanisms that appear to be responsible to influence both, including cell surviving gene Bcl-2 and BAX gene deletion and overexpression, they are responsible for cell death reduction in AVPV and eliminated sex differences in AVPV volume in mice [54–57]. However, all these manipulations were failed to alter the TH neuron number in this nucleus. Beside Bcl-2 family of proteins, other pathways appear to be responsible for a dopaminergic neuron sex difference in AVPV [16].

## Conclusion

The current investigation provides the warning of possible toxic effects of exposure of BPA substitute “BPS” during early life period. Since, BPS exposure during neonatal developmental periods act as endocrine disruptors and could have serious consequences that might alter systematic organization of reproductive organs and brain’s sex specific regions leading to severe reproductive health concerns in adulthood. Future studies are required for risk assessment of bisphenols on the sexual differentiation of AVPV region of females and to uncover mechanisms through which these bisphenols affect dimorphism of AVPV region in both sexes. Thus, we proposed that BPS cannot be recommended as a safer alternative of BPA.

## Data Availability

The datasets used and/or analyzed during the current study are available from the corresponding author on reasonable request.
